# Investigation of High HIV Prevalence in Western Equatoria State — South Sudan, 2012

**Published:** 2013-06-07

**Authors:** Samson P. Baba, Ayat Jervase, Michael St. Louis, E. Kainne Dokubo, Kevin Clarke

**Affiliations:** South Sudan Ministry of Health; Div of Global HIV/AIDS, Center for Global Health; EIS officers, CDC

Data are limited on the human immunodeficiency virus (HIV) epidemic in South Sudan, which became an independent country on July 9, 2011, after decades of civil war. In 2009, estimated HIV prevalence in antenatal clinics across the 10 states that now make up South Sudan was 3.0%, ranging from zero in Northern Bahr el Ghazal to 7.2% in Western Equatoria State (WES) (1,2). A review of HIV programmatic data in February 2012 suggested consistently higher HIV prevalence in WES than in other states. Because of concerns about the high HIV prevalence and the threat of a worsening epidemic among postconflict communities, the Ministry of Health requested assistance from CDC to investigate the high HIV prevalence in WES and provide recommendations for the public health response.

A field investigation was conducted during June 10–30, 2012. The team observed and documented HIV services provided at four antenatal clinics and three voluntary counseling and testing facilities in WES. Laboratory data were reviewed and HIV testing practices were observed to verify adherence to recommended World Health Organization/United Nations Programme on HIV/AIDS HIV testing strategies (3,4). The team abstracted and analyzed HIV testing data from antenatal clinic registers and voluntary counseling and testing data collection forms to verify the reported epidemiologic data. Using standardized inquiry domains, focus group discussions and interviews were conducted with 75 stakeholders and key informants, including government and nongovernmental officials, religious leaders, community members, health-care workers, and persons living with HIV, to describe HIV risk factors in the region. Interviews were followed by observation of social interactions and cultural practices in the communities.

HIV testing procedures were determined to be in accordance with the standard two-test serial testing algorithm used in South Sudan, and test results were accurately interpreted at the sites visited. Examination of records, review of commodity storage procedures, and cross-matching of results from confirmatory laboratories raised no substantial concerns about testing and laboratory practices. Among 420 first-visit antenatal clinic attendees, HIV seropositivity was 10.7% (95% confidence interval [CI] = 8.0%–14.2%), and among 388 voluntary counseling and testing attendees, HIV seropositivity was 13.1% (CI = 10.0%–17.0%), indicating high HIV prevalence in WES. Only 8.5% (CI = 6.0%–11.9%) of voluntary counseling and testing attendees reported condom use at last sexual intercourse, with condom unavailability stated as a key barrier. The investigation also revealed a shortage of health-care workers and lack of supportive supervision in the facilities visited, limited HIV prevention services and access to HIV testing, and limited HIV care and treatment services.

Key informant interviews suggested sexual practices (i.e., multiple concurrent sexual partners, inconsistent condom use, transactional sex, and early sexual debut) as the driver of HIV transmission. When asked about factors potentially contributing to the spread of HIV in WES, interviewees reported residual effects of conflict, poverty, stigma toward persons living with HIV, increased commercial activity and road transport, and high HIV prevalence in neighboring regions of Central African Republic, Democratic Republic of Congo, and Uganda. No reports were obtained of men who have sex with men, unusual exposure to medical injections, other use of needles, scarification, cutting instruments, or practices leading to nonsexual blood or body fluid exposure.

Financial resources for HIV prevention and treatment typically have been distributed equally across all 10 states of South Sudan. To address the high HIV prevalence in WES, the state needs to be prioritized in the national HIV response. A comprehensive HIV prevention strategy is needed, including 1) ensured access to condoms; 2) prevention interventions focused on at-risk groups, especially young women and their sex partners; and 3) expanded voluntary counseling and testing services, with linkage of persons diagnosed with HIV to strengthened HIV care and antiretroviral treatment services. Expanded surveillance also is needed to fully characterize the HIV epidemic in South Sudan.
